# Predictors of post-partum family planning uptake in Webuye Hospital, western Kenya

**DOI:** 10.4102/phcfm.v10i1.1567

**Published:** 2018-06-21

**Authors:** Henry O. Owuor, Patrick M. Chege, Jeremiah Laktabai

**Affiliations:** 1 Department of Family Medicine, Moi University, Kenya

## Abstract

**Background:**

A short inter-pregnancy interval increases the risk for maternal and neonatal deaths in addition to other pregnancy complications including: preterm delivery, low birth weight, anaemia and premature rupture of membranes. However, only one half of Kenyan women, who have no desire to conceive immediately after birth, are using contraception one year after delivery.

**Aim:**

The aim of this study was to determine the predictors of uptake of post-partum family planning (PPFP).

**Setting:**

The study was conducted among post-partum women accompanying their children for their first measles vaccination at Webuye County Hospital (WCH), in western Kenya.

**Methods:**

This was a cross-sectional study involving 259 randomly sampled post-partum women, accompanying their children for their first measles vaccination. A structured, interviewer-administered questionnaire was used to collect data. Logistic regression was used to identify correlates of PPFP uptake.

**Results:**

The uptake of PPFP among women at 9 months post-partum at WCH was found to be 78.4% ± 5.0%. The odds of PPFP uptake among women living with their sexual partners was 88.2% less than among those not living with their partners with the true population effect between 97% and 51% (OR = 0.118; 95% CI: 0.028–0.494; *p* = 0.003).

**Conclusions:**

Not living with her sexual partner in the same house is the key predictor of a woman’s PPFP uptake in WCH. This study recommends that any programme aimed at improving post-partum contraceptive use in WCH should target women who live with their partners in the same house.

## Introduction

Closely spaced pregnancies within the first year in the post-partum period are the riskiest for mother and baby, resulting in increased risks for adverse outcomes such as preterm deliveries, low birth weight and small for gestational age.^1^ Other risks associated with short inter-pregnancy intervals include neonatal death, maternal death, anaemia and premature rupture of membranes.^1,2^ The risk of child mortality is highest for very short birth-to-pregnancy intervals (<12 months) such that if all couples waited 24 months to conceive again, under-five mortality would decrease by 13%, and if couples waited 36 months, the decrease would be 25%.^3^ Family planning (FP) can avert more than 30% of maternal deaths and 10% of child mortality if couples space their pregnancies more than 2 years apart.^4^ Family planning is, therefore, recognised globally as a key life-saving intervention for mothers and their children.^5^

The prevention of unintended pregnancy and prevention of closely spaced pregnancies especially in the first 12 months following childbirth, a practice christened post-partum family planning (PPFP), should be an integral part of an existing maternal and child health and FP programme.^6^ The continuum of points of contact within the health care system that can provide opportunities for provision of integrated PPFP services include during pregnancy, labour and delivery, pre-discharge (0–48 hours), post-partum and during infant care from six weeks through 12 months.^6,7^

There is a high unmet need for FP among post-partum women. It is estimated that 222 million women in lower-income regions of the world want to avoid a pregnancy, but they use either a low-efficacy family-planning method or no method at all.^8^ According to an analysis of demographic and health survey data from 27 countries, 95% of women who are 0–12 months post-partum wanted to avoid a pregnancy in the next 24 months but 70% of them were not using any form of contraception.^9,10^ In Kenya, a study reported that 36% of post-partum women had an unmet need for FP to prevent births and 48% had an unmet need for birth spacing with an overall post-partum unmet need of 46%.^11^ This unmet need for FP among post-partum women nearly triples the 18% unmet need among married women in the country as documented by the 2014 Kenya Demographic Health Survey (KDHS).^12^

Post-partum family planning uptake remains low in Kenya and in Bungoma County in western Kenya in particular.^11,12^ This low uptake of PPFP increases the risk of unplanned early pregnancies, pregnancy losses, low birth weight, preterm deliveries and maternal and neonatal mortality even among mothers who have had multiple contacts with health facilities for other reasons other than FP. This is evidenced by the high infant and child mortality in Kenya, that is, 39 and 52 deaths per 1000 births, respectively.^12^ This study’s objective was, therefore, to determine the predictors of uptake of PPFP among post-partum women escorting their children for the first measles vaccination at Webuye County Hospital (WCH), western Kenya, so as to understand the potential barriers and facilitators of PPFP uptake. Knowledge of barriers and facilitators of PPFP will be useful in modifying practice among the health care workers who provide PPFP services at the maternal, neonatal and child health (MNCH) clinics in WCH so as to increase PPFP uptake in the facility.

## Research methods and designs

### Study design

A cross-sectional study design was used to conduct the study.

### Setting

Webuye County Hospital is a district hospital in the western region of Kenya with a catchment population of 98 494 people and 21 669 women of reproductive age (15–49 years) as stated in its annual operating plan for 2015–2016 financial year. It provides both basic and emergency obstetric and new-born care (BEmONC and CEmONC) services in addition to antenatal care (ANC), postnatal care (PNC) and child wellness clinic (CWC) for immunisations. There are about 1700 new and 4000 revisits to the ANC, and an average of 3000 deliveries annually.

### Study population and sampling strategy

The target population was post-partum women attending child immunisation clinics at Webuye Hospital. A modified systematic random sampling method was utilised to recruit study participants as there was no formal booking record at the hospital’s child wellness or immunisation clinic for mothers accompanying their babies for the nine-month measles vaccine from which to construct a sampling frame.

### Sample size determination

The sample size for the study was determined using the formula for estimation of single population proportion with the assumption of 95% confidence level, margin of error of 5% and expected prevalence of PPFP in Kenya of 51.1%.^11^ To compensate for non-response, an additional 10% were sampled. This study, therefore, recruited 259 study participants.

### Inclusion and exclusion criteria

All women who brought their children for their first measles vaccination before a year had lapsed since delivery, and consented to participate in the study, were eligible for recruitment. By this time, the mother had already had multiple contacts with the health care system, and because of weaning of the child, the protection conferred by lactational amenorrhea method (LAM) had waned. Women who attended the immunisation clinic for other vaccinations apart from the first measles vaccine, those who came for the first measles vaccine but after one ear from delivery women who were not the biological mothers of the children brought for the first measles vaccine before the first year and those who were not fit to respond adequately to questions asked owing to either the health condition of the mother or that of the child were excluded from the study.

### Pretesting the research instruments

A pretest was conducted in a different hospital before the commencement of the main study in order to establish reliability and validity of data collection instruments and research procedures.

### Data collection

A pretested, structured, interviewer-administered questionnaire was administered to the study participants by a trained research assistant. The filled questionnaires were checked daily for completeness, then coded and the data compiled in an excel database.

### Data analysis

The database was cleaned and the data exported to SPSS-IBM version 20 for analysis. Descriptive statistics were used to summarise the data. Univariate regression was used to test any associations and to identify significant determinants of PPFP uptake. Multiple logistic regression models were then used to test any significant associations between the predictor factors and uptake of PPFP methods because the outcome data collected were categorical or binary.

## Ethical considerations

This study was approved by the Moi Teaching and Referral Hospital’s Institutional Research and Ethics Committee (IREC) and was granted a formal approval number FAN: IREC 1663 on 30 June 2016.

## Results

A total of 259 women were enrolled in the study (see Table 1).

**TABLE 1:** Demographic characteristics.

Variable	Descriptive statistic
Age	27.1 ± 5.8 years
**Age group**	
15–24	37.8%
25–34	48.3%
35–44	13.5%
< 15 and > 44	0.4%
**Marital status**	
Married	86.9%
Single	12.0%
Widow or separated or divorced	1.2%
**Years of formal education**	**10.3 ± 3.3 years**
None	0.4%
Primary (1–8)	35.9%
Secondary (9–12)	42.9%
Tertiary (> 12)	20.8%
**Total number of pregnancies**	**2.6 ± 1.7 pregnancies**
1–4	86.1%
> 4	13.9%
**Living children**	**2.5 ± 1.6 children**
Male	1.2 ± 1.1
Female	1.3 ± 1.2

Majority of those who desired future conception (65.3%) were planning to wait for an average of 4 years, whereas 30.9% reported to have reached the end of their obstetric careers. More than half (57.9%) of all the women sampled reported past use of an FP method with 22.7% of these being on long-acting reversible contraceptives (LARCs) whilst the rest were on the less effective natural, hormonal or barrier FP methods. The main reason given by the mothers for unplanned pregnancies was non-use of FP (76.3%).

Contact with a formal health facility during the latest pregnancy was fairly high with 99.6% attending an average of 4 ± 1.4 ANC clinic visits, 80.7% delivering in a health facility and all the women (100%) attending the CWC. Utilisation of PNC services was; however, low at 31.7%. Of those attending ANC, only 15% were counselled on FP. Counselling rates were higher at delivery (74.2%), at PNC (72.6%) and at CWC (90.3%). Male partner involvement was high: 80% lived together with their partners and 81.7% had discussed FP. Of the women on FP, 89% reported that their partners know they are on FP; 86% of which approve the FP choice of their partners and 77% gave some form of support for the woman to get her FP of choice.

The uptake of PPFP among women at 9 months post-partum at Webuye Hospital is 78.4% ± 5.0%, and 33.8% ± 6.5% of these women are on LARCs. Three-fifths of the women who are not on PPFP are planning to have a contraceptive method (Table 2).

**TABLE 2:** Post-partum family planning utilisation in Webuye County Hospital.

Variable	Descriptive statistic
Current FP utilisation	78.4%
Planning to use if not on FP	60%
**Barriers to using FP**	
Social barriers	13.7%
Side effects	22.7%
Others	63.6%
**Method planned to use**	
Hormonal contraception ± barrier methods	48.5%
Long-acting reversible methods	24.2%
Permanent methods	3.0%
Other	24.3%
**Duration to uptake of FP**	
0–3 months	81.8%
> 3 months	6.1%
Do not know	12.1%
**Current FP choice**	
Hormonal contraception ± barrier methods	63.2%
Long-acting reversible methods	33.8%
Permanent methods	2.5%
Other	0.5%
Women’s preference	96.9%

FP, family planning.

On univariate logistic regression, the following factors were found to have significant association with PPFP uptake in Webuye Hospital:

- age (*p* = 0.002)
- marital status (*p* = 0.001)
- total number of previous pregnancies (*p* = 0.024)
- number of living children (0.011)
- having plans for additional children (*p* = 0.018)
- previous FP use before the latest pregnancy (*p* < 0.001)
- provision of FP services during the child wellness or immunisation clinics (*p* = 0.023)
- the woman living together with the partner in the same house (*p* < 0.001)
- discussing FP with the partner (*p* < 0.001)
- the male partner’s approval of FP (*p* = 0.003)
- any support offered by the male partner (*p* < 0.001).

However, only living with the partner (*p* = 0.003) remained statistically significant on multiple logistic regression as shown in Table 4. The odds of PPFP uptake among women living with their sexual partners were 88.2% less than among those not living with their partners (i.e. those not living with their partners are 8.5 times more likely to use PPFP than those living with their partners) with the true population effect between 51% and 97%. Therefore, women who live with their partners in the same house are less likely to use PPFP. There was a significant increase in current FP use to the FP prevalence before the last pregnancy (*p* < 0.001).

**TABLE 3:** Univariate logistic regression of predictive factors on post-partum family planning use in Webuye County Hospital.

S. No.	Variable	*β*	s.e.	Sig.	OR (e^β^)	95% CI for OR
1.	Age	0.090	0.029	0.002	1.094	1.033–1.159
2.	Marital status	−2.085	0.410	0.001	0.124	0.056–0.278
3.	Education (YFE)	0.029	0.045	0.527	1.029	0.941–1.125
4.	**Total pregnancies**	**0.241**	**0.106**	**0.024**	**1.272**	**1.032–1.567**
	>4	−0.609	0.508	0.230	0.544	0.201–1.471
5.	**Number of living children**	**0.301**	**0.119**	**0.011**	**1.351**	**1.071–1.705**
	Male	0.266	0.156	0.088	1.305	0.961–1.773
	Female	0.319	0.155	0.039	1.376	1.016–1.863
6.	**Plans for another child**	**-**	**-**	**0.060**	**-**	**-**
	Yes	−0.902	0.381	0.018	0.406	0.192–0.856
	Maybe or do not know	−0.560	0.860	0.515	0.571	0.106–3.802
7.	Number of future children	−0.336	0.237	0.156	0.715	0.449–1.136
8.	Duration to wait	−0.094	0.102	0.359	0.911	0.746–1.112
9.	Previous FP use	1.367	0.322	<0.001	3.925	2.089–7.374
10.	ANC attendance	−19.925	40193	1.000	0.000	0.000
11.	Number of ANC visits	0.089	0.109	0.419	1.093	0.882–1.354
12.	ANC at a PH	-	-	0.844	-	-
13.	Delivery in a PH	-	-	0.816	-	-
14.	PNC attendance	0.077	0.327	0.813	1.081	0.569–2.052
15.	FP counselling at ANC	−0.260	0.402	0.518	0.771	0.350–1.697
16.	FP services at delivery	0.386	0.359	0.281	1.472	0.729–2.972
17.	FP services during PNC	0.886	0.550	0.107	2.424	0.825–7.125
18.	FP services during CWC	1.002	0.440	0.023	2.725	1.150–6.465
19.	Counselling on spacing	−0.219	0.367	0.551	0.803	0.391–1.649
20.	Counselling on availability of FP	0.499	0.452	0.269	1.647	0.680–3.990
21.	Living with the partner	2.057	0.347	<0.001	7.826	3.964–15.452
22.	Discussing with partner	1.959	0.354	<0.001	7.092	3.542–14.200
23.	Approval by partner	1.410	0.468	0.003	4.097	1.637–10.253
24.	Support by partner	1.740	0.387	<0.001	5.700	2.670–12.166

FP, family planning; ANC, antenatal care; PNC, postnatal care; CWC, child wellness clinic; *β*, the coefficient for the constant; s.e., the standard error around the coefficient for the constant; Sig., *p*-value; OR(e^β^), the degree of association between the outcome and the predictor variables; CI, confidence interval; YFE, years of formal education; PH, public hospital; S.No. , serial number.

**TABLE 4:** Multiple logistic regression of predictors of post-partum family planning use in Webuye County Hospital.

Variable	*β*	s.e.	Sig.	OR (e^β^)	95% CI for OR
Age	−0.010	0.063	0.869	0.990	0.875–1.119
**Marital status**			0.014		
Single	−22.327	21672.2	0.999	0.000	0.000
Separated or divorced	−18.008	21672.2	0.999	0.000	0.000
Total pregnancies	0.005	0.556	0.993	1.005	0.338–2.989
Number of living children	−0.107	0.612	0.861	0.898	0.270–2.983
Female children alive	0.111	0.343	0.746	1.118	0.570–2.190
**Plans for future child**			0.690		
Yes	−18.020	15122.1	0.999	0.000	0.000
Maybe or I do not know	−18.610	15122.1	0.999	0.000	0.000
FPb4(1)	−0.583	0.598	0.330	0.558	0.173–1.803
CWCFP (1)	−0.007	0.870	0.993	0.993	0.181–5.460
Live with partner	−2.135	0.730	0.003	0.118	0.028–0.494
Discussed with partner	−0.314	0.857	0.714	0.731	0.136–3.915
Partner approval	−1.975	1.098	0.072	0.139	0.016–1.194
Partner support	0.152	1.063	0.887	1.164	0.145–9.353

FP, family planning; CWCFP, child wellness clinic family planning; *β*, the coefficient for the constant; s.e., the standard error around the coefficient for the constant; Sig., *p*-value; OR(e^β^), the degree of association between the outcome and the predictor variables; CI, confidence interval.

## Discussion

### Key findings

The uptake of PPFP among women at 9 months post-partum at Webuye Hospital is 78.4% ± 5.0%, and 33.8% ± 6.5% of these women are on LARCs. There was a significant increase in current FP use to the FP prevalence before the last pregnancy (*p* < 0.001). Living with the sexual partner was the only independent variable that was significantly associated with PPFP uptake (OR = 0.118; 95% CI: 0.028–0.494; *p* = 0.003). That is to say, the odds of PPFP uptake among women living with their sexual partners was 88.2% less than among those not living with their partners (i.e. those not living with their partners are 8.5 times more likely to use PPFP than those living with their partners) with the true population effect between 97% and 51%.

### Discussion of key findings

The level of current use of PPFP (contraceptive prevalence rate) among women taking their children for measles vaccination at 9 months in WCH is much higher than the national contraceptive prevalence rate (CPR) that is 58%.^12^ The CPR for Bungoma County and western region is 55.5% and 58.6%, respectively.^12^ In addition, the uptake of the LARCs in this study (33.8%) is also higher than the national average of 13.3%.^12^ The prevalence of LARCs use in Bungoma County and western region is 12.6% and 16.5%, respectively.^12^ The higher prevalence rate or uptake of FP in the sampled population may be explained by the differing characteristics of the sample population and the high health service contact it has compared to other women in Bungoma County and the western region in general. In addition, this was a facility-based study, whereas the KDHS was a community-based study.

The literacy levels and the average age of the sample population may be particularly contributive to the higher prevalence or uptake of FP than in the general population. Level of education, socio-economic status and age of the woman were identified as factors that influence the intention to use PPFP in Nigeria.^13,14^ Higher levels of education result in a better understanding of the available modern contraceptive methods, the benefits of fertility regulation, awareness of the side effects of contraceptive methods and preference for the most convenient ones.^15^ PPFP uptake is higher among women in the 20–29 age categories because they have not finished child-bearing.^13^ In addition, the women in this age category have a higher intention to use a contraceptive method.^13^ In the KDHS, 42.7% of women had at least secondary education, whereas 37.2% were below 25 years of age.^12^

The significant increase in current FP use to the FP prevalence before the latest pregnancy may be explained by the higher counselling rates and FP service provision during the multiple interactions with the health facility, for example, during MNCH clinic visits.^16,17^

The only factor that was predictive of increased PPFP uptake was not living with the sexual partner in the same house. Women who lived with their partners were less likely to use FP than those who do not. Men often make the final decision about FP use, especially in traditional patriarchal societies like in Kenya.^15,18^ Women who live with their sexual partners may be highly dependent on their spouses for approval of modern contraceptive use. On the contrary, women who do not live with their sexual partners may be under no obligation to seek approval from any one concerning contraceptive use. Thus, partner’s non-approval may be a major reason for non-use of modern contraceptive among the women who live with their sexual partners in the same house.^19^ Studies have shown that male dominated decision-making about fertility preference is associated with lower use of contraceptives.^20,21,22,23,24^ On the contrary, other studies have revealed that women who live or cohabit with a partner use more FP services than those who live separately from their partners.^25^ This has been linked to the support the women get from their partners.

The lack of association between PPFP uptake and ANC attendance may be attributed to the timelag between the counselling, the need for the contraceptive,^26^ and the absence of provision of FP information and services during ANC.^27^ Other studies have found that prenatal contraceptive counselling may benefit women who are economically disadvantaged and with a lower education level than those who are more educated, suggesting an interaction with education level of the women.^28^ The lack of association between facility delivery and PPFP uptake in this study could be because of lack of simultaneous provision of FP counselling and FP method to the woman before discharge from the hospital.

Other factors that have been associated with the uptake of FP like age, the number of living children or previous pregnancies, previous FP use, plans for additional children, discussing FP with the partner and partner’s approval and support were not associated with PPFP uptake in this population. The lack of association between these factors and PPFP uptake may be masked by the effect of integrating FP services into the MNCH services, the high utilisation of health services in this population and the demographic characteristics.

### Strengths and limitations

Recall bias and social desirability bias are all inherent weaknesses of cross-sectional studies. These were; however, minimised by assuring the study participants of confidentiality and interviewing them in private areas within the clinic in the absence of a third party. The social distance was also minimised. The study was conducted in a single facility, but findings are generalisable to other facilities in the region or to the population in this geographical area because the measles vaccine coverage in Bungoma is quite high at 84.3%.^12^

### Implications or recommendations

With these findings, the study recommends that programmes aimed at enhancing post-partum contraceptive use in WCH should target women who live with their partners in the same house, as they are less likely to use PPFP. Increasing reproductive health education among post-partum women who live with their partners in the same house will significantly improve the uptake of PPFP in WCH. This study further recommends that the national and/or county government and development partners to sponsor training of FP providers, MCH nurses and other care providers on the role of the marital status of the post-partum woman and her living with her partner factors to bolster PPFP use in WCH.

## Conclusion

The PPFP uptake in WCH is higher than both the national and county contraceptive prevalence rates. Not living with the sexual partner in the same house is the key predictor of a woman’s PPFP uptake in WCH. The observed integration of FP with MNCH services should be strengthened as this may have contributed to the increase in FP use at 9 months post-partum compared to FP use before the latest pregnancy.
